# Secondary nuclear targeting of mesoporous silica nano-particles for cancer-specific drug delivery based on charge inversion

**DOI:** 10.18632/oncotarget.12149

**Published:** 2016-09-20

**Authors:** Jianwen Zhao, Fengfeng Zhao, Xiyong Wang, Xiaobo Fan, Guoqiu Wu

**Affiliations:** ^1^ Center of Clinical Laboratory Medicine of Zhongda Hospital, Southeast University, Nanjing, 210009, China; ^2^ Medical School, Southeast University, Nanjing, 210009, China

**Keywords:** peptide charge inversion, secondary nuclear targeting, mesoporous silica, cancer, delivery

## Abstract

A novel multifunctional nano-drug delivery system based on reversal of peptide charge was successfully developed for anticancer drug delivery and imaging. Mesoporous silica nano-particles (MSN) ~50 nm in diameter were chosen as the drug reservoirs, and their surfaces were modified with HIV-1 transactivator peptide-fluorescein isothiocyanate (TAT-FITC) and YSA-BHQ1. The short TAT peptide labeled with FITC was used to facilitate intranuclear delivery, while the YSA peptide tagged with the BHQ1 quencher group was used to specifically bind to the tumor EphA2 membrane receptor. Citraconic anhydride (Cit) was used to invert the charge of the TAT peptide in neutral or weak alkaline conditions so that the positively charged YSA peptide could combine with the TAT peptide through electrostatic attraction. The FITC fluorescence was quenched by the spatial approach of BHQ1 after the two peptides bound to each other. However, the Cit-amino bond was unstable in the acidic atmosphere, so the positive charge of the TAT peptide was restored and the positively charged YSA moiety was repelled. The FITC fluorescence was recovered after the YSA-BHQ1 moiety was removed, and the TAT peptide led the nano-particles into the nucleolus. This nano-drug delivery system was stable at physiological pH, rapidly released the drug in acidic buffer, and was easily taken up by MCF-7 cells. Compared with free doxorubicin hydrochloride at an equal concentration, this modified MSN loaded with doxorubicin molecules had an equivalent inhibitory effect on MCF-7 cells. This nano-drug delivery system is thus a promising method for simultaneous cancer diagnosis and therapy.

## INTRODUCTION

Chemotherapy has greatly increased the survival rates of patients with cancer, but traditional chemotherapeutic drugs lack specificity and thus damage healthy cells/tissues, resulting in serious side-effects that immensely reduce the therapeutic effects [1–3]. Recently, nano-medicine carrier systems that actively and passively target cancerous cells have provided a promising alternative for the early diagnosis and treatment of cancer, due to their distinct pharmacokinetics and biological distribution [4, 5]. The specific targeting and pharmacology of nano-particles could be improved by means of a decorating targeting segment on the surface, such as a vitamin, antibody, protein, aptamer, or peptide. This active targeting strategy could vastly improve the antineoplastic effects of anticancer drugs and increase the imaging sensitivity of tumor tissues by enhancing the signal-to-noise ratio [6–9].

It has been reported that the drug release from carrier systems occurs mainly in the cytoplasm, so the maximal anticancer effects cannot be achieved against targets inside the cell nucleus [10]. One solution to this has been to incorporate a nuclear localization signal (NLS) peptide into the carrier system, such as simian virus 40 T-antigen (SVT) or human immunodeficiency virus 1 transactivator protein (HIV TAT) [11–13]. The physicochemical properties, targeting potential and transposition mechanisms of these NLS peptides have been clearly demonstrated. NLS peptides first form a complex with importin α on the karyotheca. Then, importin β is recruited to the complex, and the NLS peptides are conveniently transported into the nucleus [14–16]. However, NLS peptides can cause serious serum inhibition and are subject to plasma clearance because they are highly positively charged. The TAT peptide, for example, possesses a highly cationic motif of nine basic amino acids (KKKRRQRRR) [17–19]. Moreover, these peptides are usually nonspecific for tumor cells and thus poison normal cells as well. Therefore, single cell-membrane- or nuclear-receptor-targeting peptides are not the best choice for the decoration of nano-particles for drug delivery [17–20]. To solve all the above-mentioned problems, our research group designed a drug delivery system based on peptide charge inversion, in order to achieve continuous multistage targeting and simultaneously perform fluorescent imaging and nuclear drug delivery.

Mesoporous silica nano-particles (MSN) are among the most promising drug-delivery vehicles, and have been used to fabricate controlled drug-release systems due to their remarkable properties, including easy synthesis, large pore volume, modifiable surface, uniform morphology, high biochemical and physicochemical stability, easy functionalization, good biocompatibility and very low cytotoxicity [21–26]. In addition, the vast numbers of original silanol groups (Si-OH) on MSN facilitate their post-functionalization [21, 24, 26]. Moreover, the US Food and Drug Administration has permitted the use of silicon nano-particles for biomedical applications [27]. Thus, we chose to use commercial MSN formed by a calcination process with a diameter of ~50 nm as drug reservoirs, because the cellular nuclear pore complexes permit the active transport of particles 20–70 nm in size [14–16, 28, 29].

## RESULTS AND DISCUSSION

### Characterization of MSN/COOH/TAT-FITC/Cit/YSA-BHQ1/DOX

**Scheme 1:**
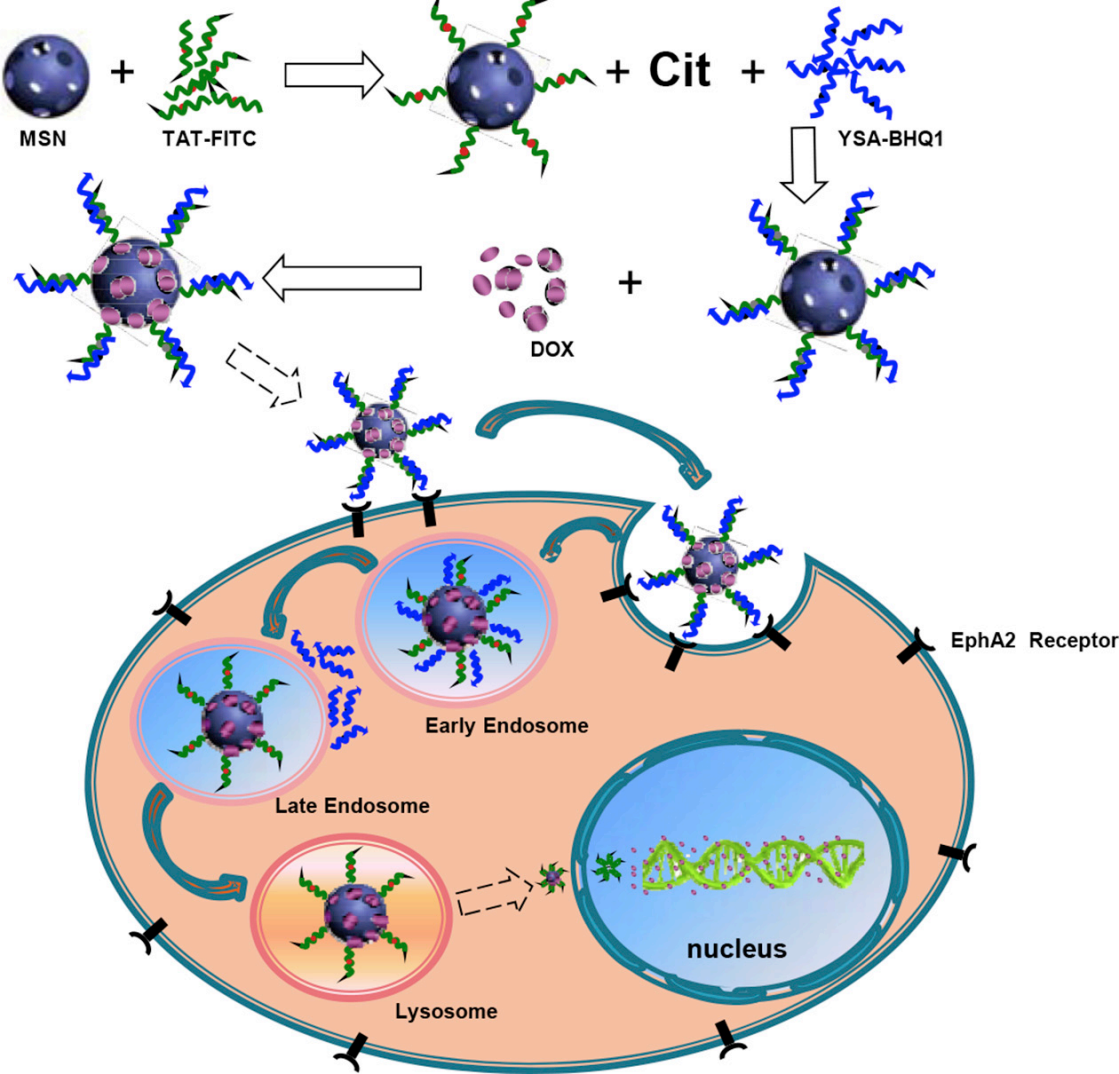
Illustration of the synthesis and mechanism of action in MCF-7 cells of MSN/COOH/TAT-FITC/Cit/YSA-BHQ1/DOX

**Figure 1:**
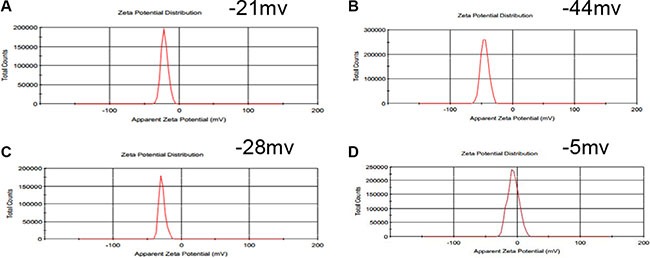
The zeta potentials of MSN (A), MSN/COOH (B), MSN/COOH/TAT-FITC (C), and MSN/COOH/TAT-FITC/Cit/YSA-BHQ1 (D)

**Figure 2:**
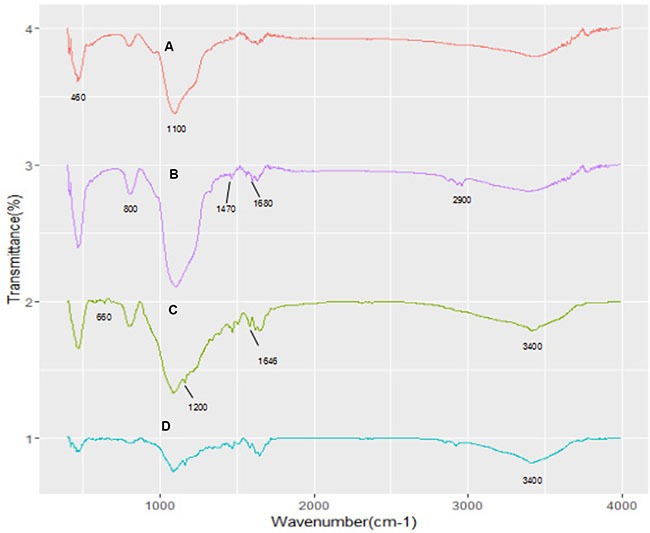
FT-IR spectra of MSN (A), MSN/COOH (B), MSN/COOH/TAT-FITC (C), and MSN/COOH/TAT-FITC/Cit/YSA-BHQ1 (D)

**Figure 3:**
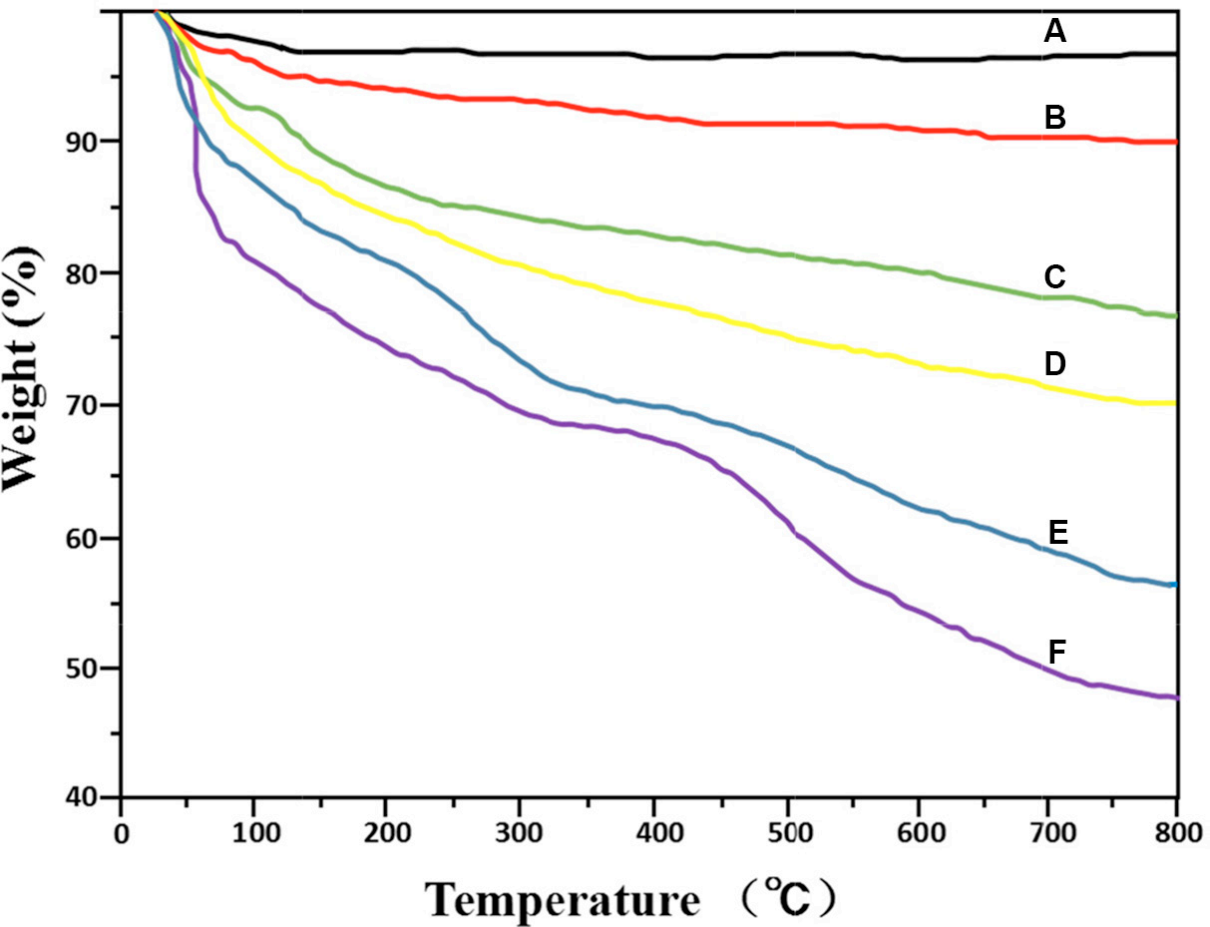
Thermogravimetric analysis curves of MSN. (**A**), MSN/COOH (**B**), MSN/COOH/TAT-FITC (**C**), MSN/COOH/TAT-FITC/Cit (**D**), MSN/COOH/TAT-FITC/Cit/YSA-BHQ1 (**E**), and MSN/COOH/TAT-FITC/Cit/YSA-BHQ1/DOX (**F**)

The synthesis procedure of MSN/COOH/TAT-FITC/Cit/YSA-BHQ1/DOX is illustrated in Scheme 1. The functionalization of MSN was performed by a three-step superficial embellishment process. Firstly, the hydroxy groups on the MSN reacted with CPTS to form MSN/COOH, which made the surfaces of the nano-particles more negative. As illustrated in Figure 1, the zeta potential of MSN/COOH was lower than that of crude MSN. The FT-IR spectrum of MSN/COOH (Figure 2B) displayed peaks at 2900 cm^−1^ and 1470 cm^−1^, corresponding to the stretching vibration and bend vibration of O-H, respectively. The peak at 1680 cm^−1^ was due to the stretching vibration of C = O [30, 31]. Thermogravimetric analysis was used to estimate the grafting ratio of the surface modifications on the MSN. As demonstrated in Figure 3, the unmodified MSN (curve a) lost about 2 wt% before 120°C, which was attributed to the elimination of adsorbed water on the surface [32]. As shown in Figure 3B, 9 wt% weight loss occurred at 800°C. With consideration of the 2 wt% for adsorbed water on the MSN, it can be estimated that the content of CPTS on the MSN was about 7 wt%.

**Figure 4:**
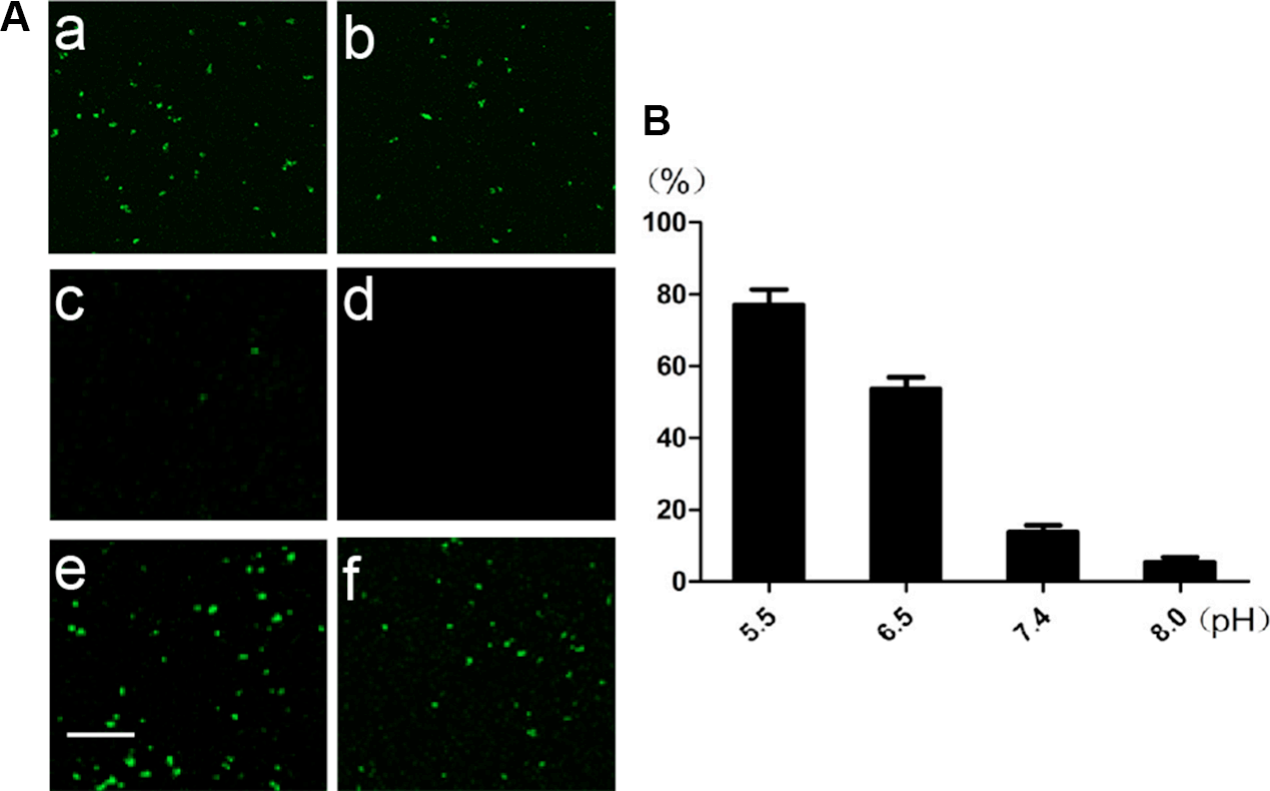
(**A**) Images of MSN/COOH/TAT-FITC/Cit/YSA-BHQ1 under the fluorescence microscope at pH 5. 5 (a), 6.5 (b), 7.4 (c), and 8.0 (d), and images of MSN/COOH/TAT-FITC (e) and MSN/COOH/TAT-FITC/Cit (f) at pH 7.4. The scale bar is 100 μm. (**B**) The percentage of fluorescence intensity of MSN/COOH/TAT-FITC/Cit/YSA-BHQ1 at different pHs, compared with that of MSN/COOH/TAT-FITC at pH 7.4.

Second, MSN/COOH was connected with the TAT-FITC peptides. The carboxyl group of MSN/COOH reacted with the amino of the TAT-FITC peptide via amidation reaction. As illustrated in Figure 1, the zeta potential of MSN/COOH/TAT-FITC was higher than that of MSN/COOH, because the TAT peptides bore positive charge. As shown in Figure 2C, peak absorptions at 1200 cm^−1^ and 1646 cm^−1^ were apparent in the FT-IR spectrum of MSN/COOH/TAT-FITC, corresponding to the stretching vibrations of C-N and C = O, respectively. The peak at 660 cm^−1^ was attributed to the bend vibration of N-H. The stretching vibration of N-H was around 3400 cm^−1^, which overlapped with that of Si-OH [30, 31]. These results demonstrated that the TAT-FITC peptides were conjugated onto MSN/COOH. According to curve c in Figure 3, the amount of TAT-FITC that was grafted onto MSN/COOH was about 14 wt%. MSN/COOH/TAT-FITC could be visualized by the green color of FITC under the fluorescence microscope (Figure 4).

**Figure 5:**
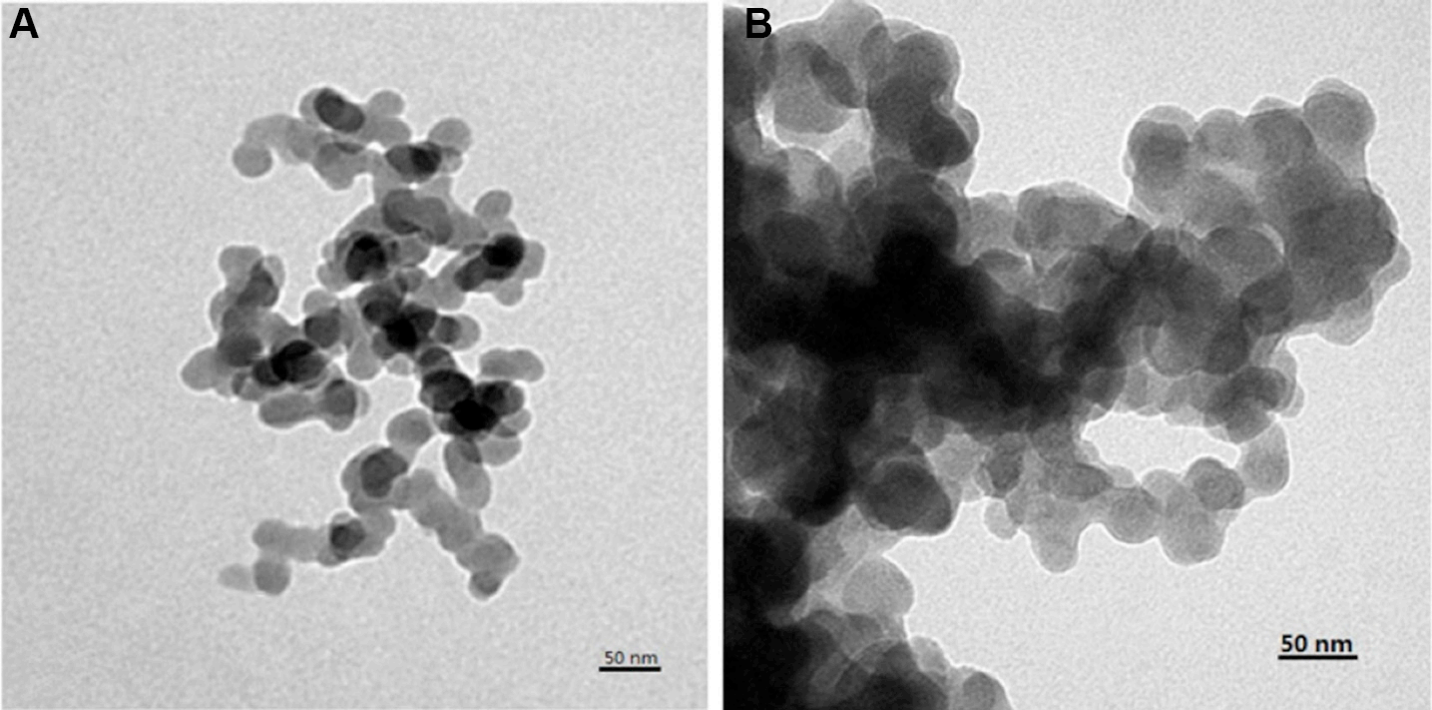
TEM images of MSN (A) and MSN/COOH/TAT-FITC/Cit/YSA-BHQ1 (B)

Finally, the YSA-BHQ1 peptides were combined with MSN/COOH/TAT-FITC through electrostatic interaction after the charge reversal of the TAT peptides. The sequence of the YSA peptide was derived from the EphA2-binding YSA peptide identified by phage display, which has been reported as an ephrin mimetic peptide that specifically binds to the EphA2 receptor [33]. EphA2 receptors are overexpressed in many types of carcinoma cells and tumor blood vessels, while they are not expressed in quiescent vasculature and are expressed at low levels in normal tissues and cells [33–35]. The amino-terminal portion of the YSA peptide appears to be the most important for EphA2 receptor binding and selectivity, while the carboxyl terminus is the region more amenable to modification without an effect on potency [36]. Thus, we tagged the carboxyl terminus of the YSA peptide with BHQ1 to avoid reducing the EphA2 binding activity [37]. The free terminal amine groups of YSA are cationic at a neutral pH [38]. As shown in Figure 1D, the zeta potential of MSN/COOH/TAT-FITC/Cit/YSA-BHQ1 was negatively charged (−5 mv). The negative charge of the nano-particles decreased, because the YSA-BHQ1 peptides were positively charged. This feature has been shown to extend the *in vivo* circulation of nano-particles in the blood vessels by slowing their clearance by the reticuloendothelial system [39]. The grafting ratios of citraconic amide and YSA-BHQ1 on MSN were about 6 wt% and 14 wt%, respectively (Figure 3D and 3E). After the loading of DOX, the amount of DOX on the MSN was estimated to be about 8 wt% (Figure 3F). Fluorescent imaging revealed that the fluorescence of MSN/COOH/TAT-FITC/Cit/YSA-BHQ1 was quenched under neutral or alkaline conditions. In contrast, MSN/COOH/TAT-FITC/Cit/YSA-BHQ1 fluoresced in an acidic environment, and the fluorescence intensity increased as the solution pH decreased (Figure 4). These results demonstrated that the YSA-BHQ1 peptides were repelled from MSN/COOH/TAT-FITC under acidic conditions. After the peptide decoration, the nano-particles were obviously larger in size than the crude MSN (Figure 5). The TEM images revealed uniform size distributions for MSN and MSN/COOH/TAT-FITC/Cit/YSA-BHQ1, with average diameters of about 35 nm and 50 nm, respectively.

**Figure 6:**
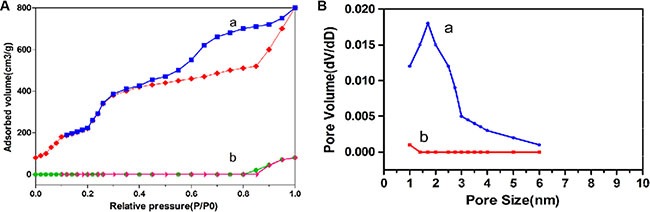
(**A**) Nitrogen adsorption-desorption isotherms of MSN (a) and MSN/COOH/TAT-FITC/Cit/YSA-BHQ1/DOX (b); (**B**) The pore size distributions of MSN (a) and MSN/COOH/TAT-FITC/Cit/YSA-BHQ1/DOX (b)

The surface areas, pore sizes, and pore volumes of MSN and MSN/COOH/TAT-FITC/Cit/YSA-BHQ1/DOX were measured by a nitrogen adsorption-desorption test. Figure 6 displays the nitrogen adsorption-desorption isotherms of the MSN and MSN/COOH/TAT-FITC/Cit/YSA-BHQ1/DOX and the pore size distributive curves derived from the adsorption branches of the nitrogen isotherms by the Barrett-Joynere-Halenda method. These two samples displayed typical IV features for mesoporous silica according to the IUPAC classification, with well-defined steps at relative pressures of 0.2–0.4*P*/*P*_0_. The MSN had a high specific surface area (496 m^2^/g) and pore volume (0.98 mL/g). However, the specific surface area and pore volume of MSN/COOH/TAT-FITC/Cit/YSA-BHQ1/DOX were reduced to 157 m^2^/g and 0.27 mL/g, respectively. This result should be largely attributed to the attachment of organic groups (i.e., TAT- FITC and YSA-BHQ1 peptides) and DOX molecules to the MSN surface or porous structures [40]. The average pore size of the MSN was 2.2 nm, while the molecular diameter of DOX was estimated to be about 1.37 nm by Chemdraw software; thus, the MSN pores were large enough to be filled by the DOX molecules [41]. However, the average pore size of MSN/COOH/TAT-FITC/Cit/YSA-BHQ1/DOX was reduced to 0.7 nm, due to the DOX molecules filling the pores.

### DOX loading and release

The loading efficiency and amount of the DOX molecules in the nano-particles were calculated by the following equations:

Loading efficiency (%, w/w) = ((DOX in feed − free DOX)/DOX in feed) × 100.

Loading amount (%, w/w) = ((DOX in feed − free DOX)/nano-particles in feed) × 100.

According to these equations, the loading efficiency and amount of the DOX molecules were 8.6 ± 1.4% and 39.3 ± 3.5%, respectively, indicating that MSN/COOH/TAT-FITC/Cit/YSA-BHQ1 could be used to effectively load DOX molecules.

**Figure 7:**
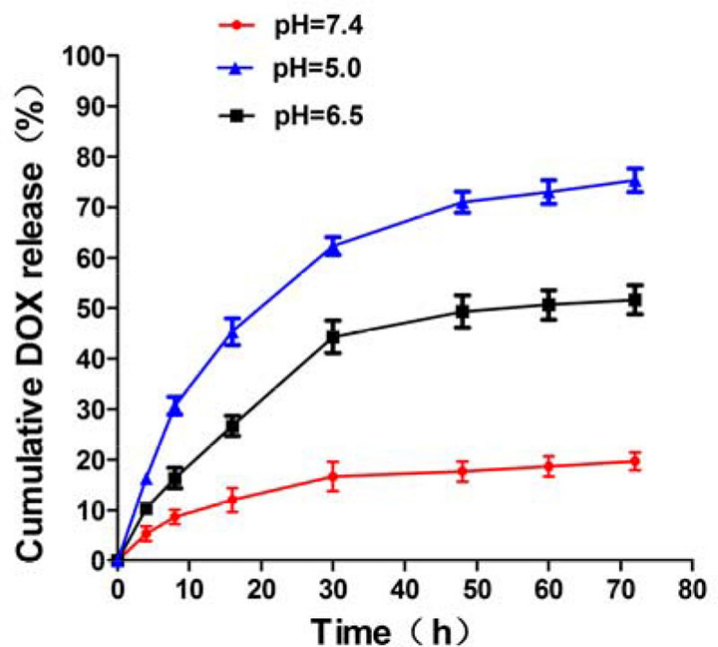
pH-dependent release of DOX molecules from MSN/COOH/TAT-FITC/Cit/YSA-BHQ1

The release patterns of DOX molecules from MSN/COOH/TAT-FITC/Cit/YSA-BHQ1 were investigated under different pH conditions. As shown in Figure 7, the release amount of DOX molecules was considerably limited; only about 10% of the DOX molecules had been released after 10 h at pH 7.4. This result indicated that the electrostatic interactions between DOX molecules and nano-particles were relatively stable under physiological pH conditions. However, an acidic atmosphere accelerated the release of DOX molecules; the release ratio reached 19% at pH 6.5 and 34% at pH 5.0 after 10 h, indicating that the electrostatic interactions between DOX molecules and nano-particles were weakened by the protonation effect. This experiment clearly demonstrated that the pH of the medium significantly influenced the quantity of DOX molecules released from the nano-particles. An explanation for this phenomenon may be that the acidic buffer weakened the electrostatic interactions between the DOX molecules and the nano-particles by neutralizing the negative charge, such that the DOX molecules were released from the charged complex. This pH-dependant release behavior of drug carriers will be beneficial in promoting the release of DOX molecules at the tumor site, where the microenvironment is acidic. Thus, MSN/COOH/TAT-FITC/Cit/YSA-BHQ1/DOX exhibited tremendous potential for use in cancer therapy.

### *In vitro* cytotoxicity studies

**Figure 8:**
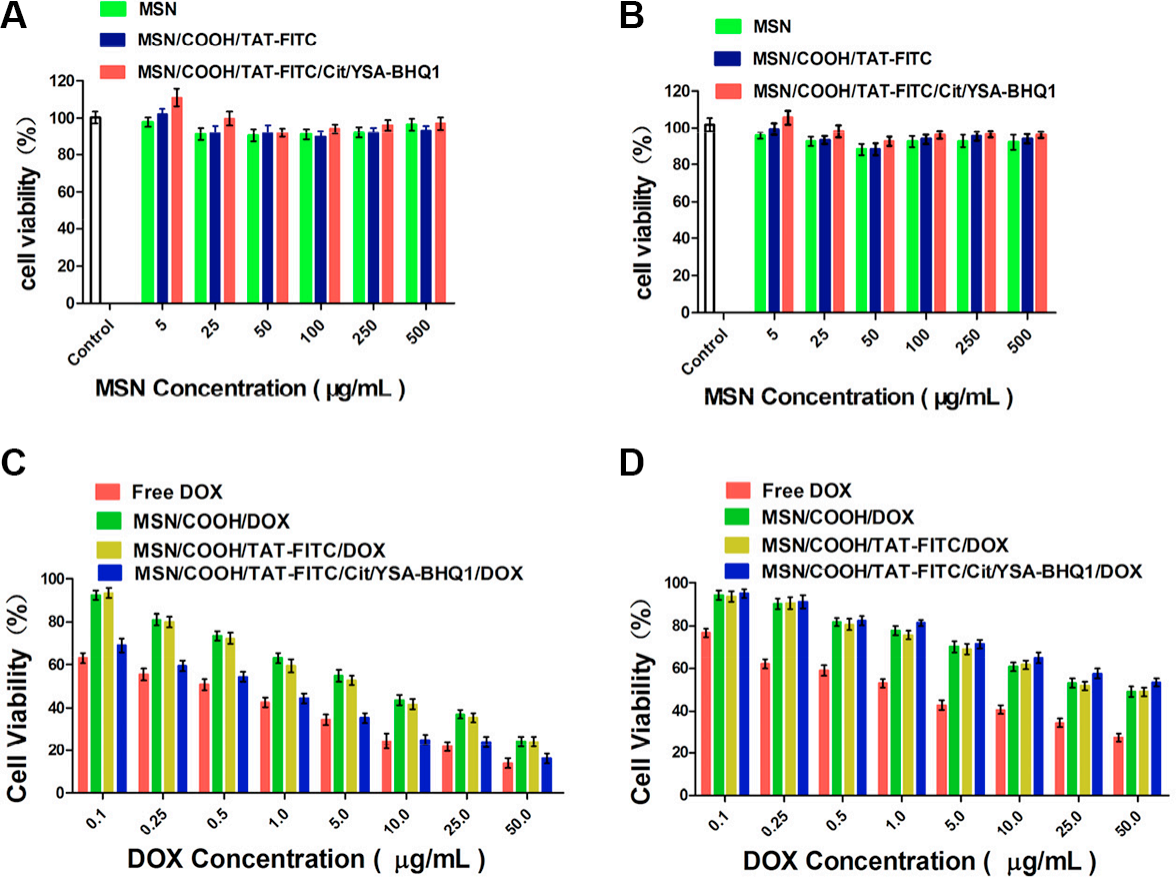
(**A** and **B**) Viability of MCF-7 and HEK293 cells after treatment with different nano-particles at 37°C for 24 h (MSN, MSN/COOH/TAT-FITC, or MSN/COOH/TAT-FITC/Cit/YSA-BHQ1 vs. control, *p* > 0.05). (**C**) Viability of MCF-7 cells after treatment with DOX-loaded nano-particles or free DOX at 37°C for 24 h (MSN/COOH/DOX or MSN/COOH/TAT-FITC/DOX vs. free DOX, *p* < 0.01. MSN/COOH/TAT-FITC/Cit/YSA-BHQ1/DOX vs. free DOX, *p* > 0.05). (**D**) Viability of HEK293 cells after treatment with DOX-loaded nano-particles or free DOX at 37°C for 24 h (MSN/COOH/DOX, MSN/COOH/TAT-FITC/DOX, or MSN/COOH/TAT-FITC/Cit/YSA-BHQ1/DOX vs. free DOX, *p* < 0.01).

MCF-7 and HEK293 cells were used to assess the *in vitro* cytotoxicity of the constructed nano-particles. As shown in Figure 8A and 8B, MSN, MSN/COOH/TAT-FITC, and MSN/COOH/TAT-FITC/Cit/YSA-BHQ1 without DOX had very limited effects on the MCF-7 and HEK293 cells at different concentrations, indicating their good biocompatibility and the very low cytotoxicity of MSN/COOH/TAT-FITC/Cit/YSA-BHQ1 as a drug vehicle. However, MSN/COOH/DOX and MSN/COOH/TAT-FITC/DOX demonstrated obvious toxicity against MCF-7 and HEK293 cells. As a positive control, free DOX displayed the strongest inhibitory effect against MCF-7 and HEK293 cells among all the groups. This was because the molecular size of DOX is about 1.37 nm [41], so DOX can easily enter into cells and accumulate. The viability of MCF-7 cells treated with the MSN/COOH/TAT-FITC/Cit/YSA-BHQ1/DOX was equivalent to that of cells treated with free DOX, indicating that the YSA peptides were beneficial for the uptake of MSN/COOH/TAT-FITC/Cit/YSA-BHQ1/DOX by MCF-7 cells via EphA2 receptor-mediated endocytosis, leading to enormous inhibition of MCF-7 cell growth. After treatment with MSN/COOH/TAT-FITC/Cit/YSA-BHQ1/DOX, the HEK293 cells had higher viability than MCF-7 cells. The main reason for this was that far fewer EphA2 receptors are expressed by HEK293 cells than by MCF-7 cells, resulting in far lower extent of receptor-mediated endocytosis. Thus, it was difficult for MSN/COOH/TAT-FITC/Cit/YSA-BHQ1/DOX to be taken up by HEK293 cells, suggesting that MSN/COOH/TAT-FITC/Cit/YSA-BHQ1/DOX had limited toxicity to normal cells.

### Cell apoptosis study

**Figure 9:**
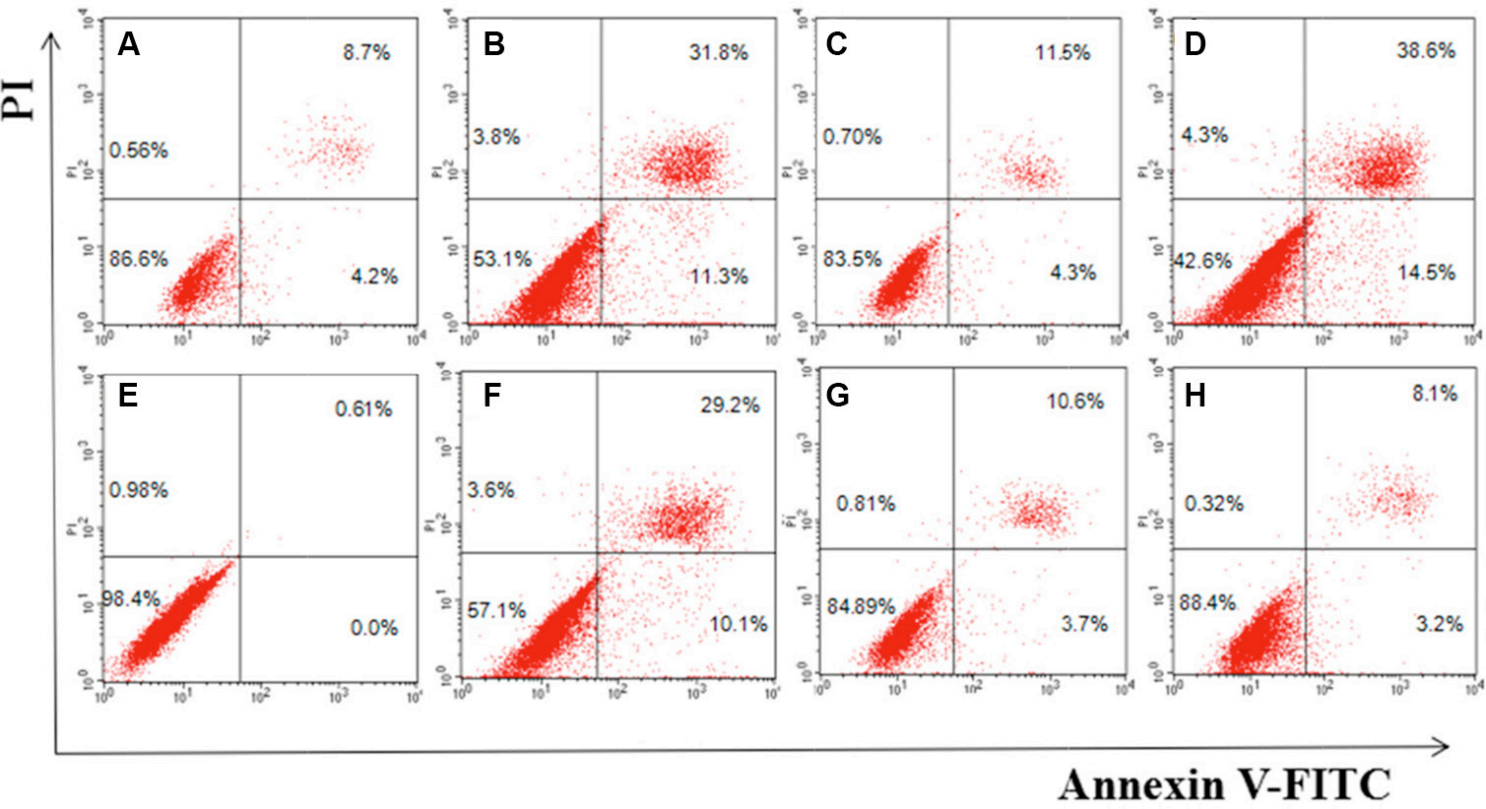
Flow cytometry analysis for apoptosis of MCF-7 (A–D) and HEK293 cells (E–H) induced by MSN/COOH/TAT-FITC/Cit/YSA-BHQ1, free DOX, MSN/COOH/DOX, or MSN/COOH/TAT-FITC/Cit/YSA-BHQ1/DOX for 24 h, respectively (Lower-right quadrant: early apoptotic cells, i.e., Annexin V-FITC-positive/PI-negative cells; upper-right quadrant: necrotic or late-apoptotic cells, i.e., Annexin V-FITC-positive/PI-positive cells).

The apoptosis of MCF-7 and HEK293 cells after treatment with free DOX, MSN/COOH/DOX, MSN/COOH/TAT-FITC/Cit/YSA-BHQ/DOX, or MSN/COOH/TAT- FITC/Cit/YSA-BHQ1 was measured with a FACSCalibur Flow Cytometer. As shown in Figure 9, the free DOX molecules strongly promoted the apoptosis of MCF-7 and HEK293 cells, due to the easy entrance of the DOX molecules into the cells. Compared with free DOX, MSN/COOH/DOX induced apoptosis to a lesser extent in MCF-7 and HEK293 cells. However, MSN/COOH/TAT-FITC/Cit/YSA-BHQ1/DOX caused obvious apoptosis of MCF-7 cells, illustrating that the YSA peptides effectively targeted the MCF-7 cells and increased the cellular uptake of the nano-particles. MSN/COOH/TAT-FITC/Cit/YSA-BHQ1/DOX had a lesser effect on the apoptotic ratio of HEK293 cells, which should be attributed to very low expression of EphA2 receptors on HEK293 cells. In addition, MSN/COOH/TAT-FITC/Cit/YSA-BHQ1 had little influence on the apoptosis of MCF-7 and HEK293 cells. This result again confirmed the safety of MSN/COOH/TAT-FITC/Cit/YSA-BHQ1.

### Intracellular uptake of nano-particles

**Figure 10:**
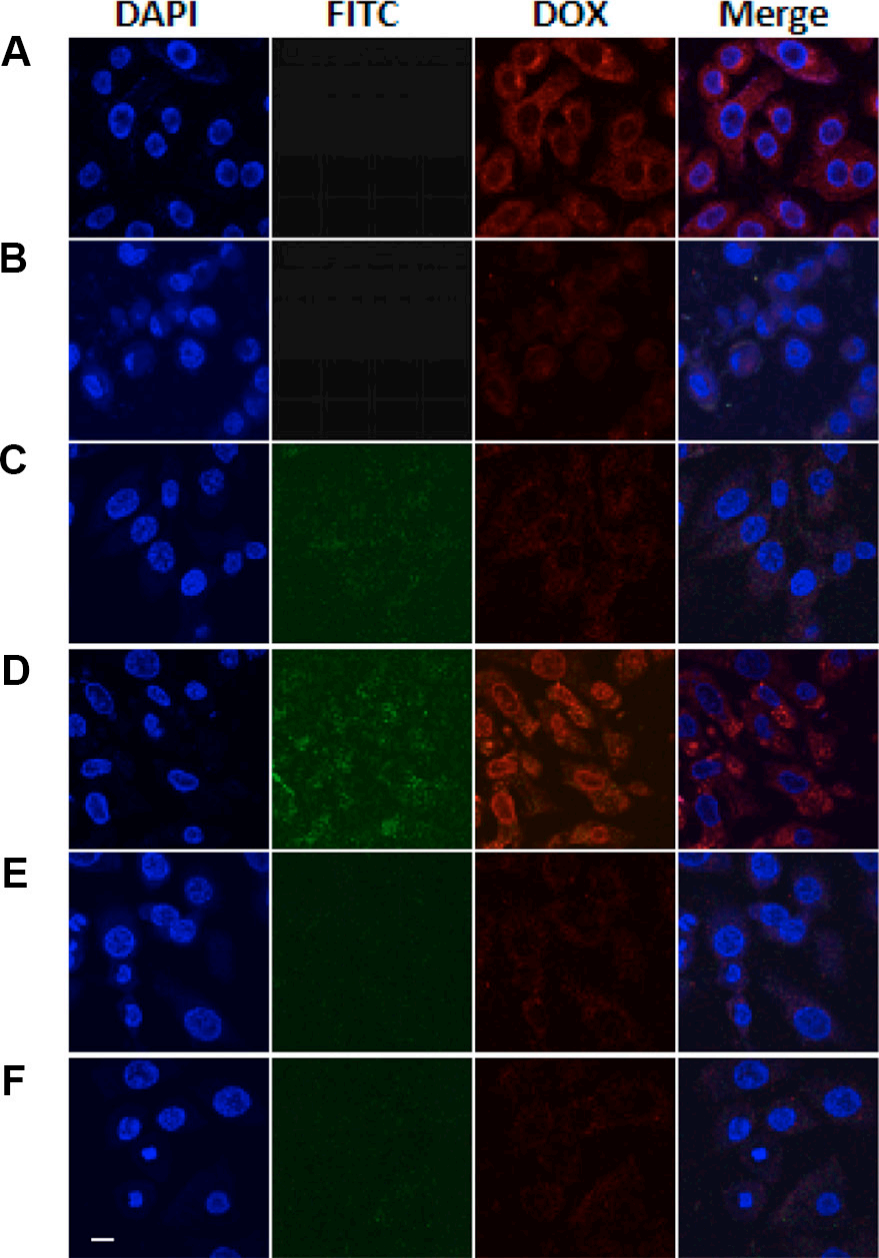
CLSM images of MCF-7 cells treated with free DOX (A), MSN/COOH/DOX (B), MSN/COOH/TAT-FITC/DOX (C) or MSN/COOH/TAT-FITC/Cit/YSA- BHQ1/DOX (D) MCF-7 cells pretreated with YSA peptides and then treated with MSN/COOH/TAT-FITC/Cit/YSA-BHQ1/DOX (**E**) and HEK 293 cells treated with MSN/COOH/TAT-FITC/Cit/YSA- BHQ1/DOX (**F**) (DOX concentration of 5 μg/mL) at 37°C for 2 h. The red, green, and blue colors were regarded as the fluorescences of DOX, FITC, and DAPI, respectively. The scale bar is 10 μm. The brightness of FITC images have been enhanced by 20%.

CLSM was used to observe the uptake of MSN/COOH/DOX, MSN/COOH/TAT-FITC/DOX, MSN/COOH/TAT-FITC/Cit/YSA-BHQ1/DOX and free DOX molecules by MCF-7 and HEK293 cells (Figure 10). The red fluorescence represents the DOX molecules, while green fluorescence represents FITC and blue fluorescence represents DAPI. As shown in Figure 10, intense red fluorescence was observed in the MCF-7 cells treated with free DOX (Figure 10A), indicating that free DOX could quickly penetrate the cytomembrane and enter into the cells [42]. However, because free DOX can non-specifically poison normal cells/tissues and cause serious side effects, its anticancer potential is limited. MCF-7 cells exposed to MSN/COOH/DOX (Figure 10B) or MSN/COOH/TAT-FITC/DOX (Figure 10C) exhibited a weak red fluorescence, indicating that a small quantity of DOX molecules had entered into the cells. Importantly, MCF-7 cells displayed strong red and green fluorescence after treatment with MSN/COOH/TAT-FITC/Cit/YSA-BHQ1/DOX (Figure 10D), indicating the abundant accumulation of nano-particles inside the cells. This was because the YSA peptides on the nano-particles could specifically bind to the EphA2 receptors overexpressed on the MCF-7 cell surface, such that nano-particle uptake was accelerated through specific receptor-mediated endocytosis. Consistent with this result, MCF-7 cells pre-treated with excess YSA peptides displayed weak red and green fluorescence after being exposed to MSN/COOH/TAT-FITC/Cit/YSA-BHQ1/DOX (Figure 10E), because the large number of EphA2 receptors occupied by free YSA peptides prohibited the receptor-mediated binding of the YSA-functionalized MSN. Weak red and green fluorescence was observed after HEK293 cells were exposed to MSN/COOH/TAT-FITC/Cit/YSA- BHQ1/DOX (Figure 10F), implying that only a small amount of MSN/COOH/TAT- FITC/Cit/YSA-BHQ1/DOX entered into the HEK293 cells. This was because EphA2 receptors are mainly expressed by tumor cells and barely present on normal cells/tissues [33, 43].

## MATERIALS AND METHODS

MSN were purchased from Xinlei Powder Technology Limited Company (Bengbu, China). N-(3-trimethoxysilylpropyl)–ethylenediamine triacetic acid trisodium salt (45% in water) (CPTS) was purchased from Maror Chemistry Technology Limited Company (Shanghai, China). DOX (purity > 99%) was obtained from Macklin Chemistry Technology Limited Company (Shanghai, China). The TAT-FITC (YGRKKKRRQRRRYK-FITC) and YSA-BHQ1 (YSAYPDSVMMSKRR- RRR-BHQ1) peptides were purchased from Shanghai Ketai Biotechnology Limited Company (Shanghai, China). N-hydroxysulfosuccinimide sodium salt (sulfo-NHS), N-(3-dimethylaminopropyl)–N-ethylcarbodiimide hydrochloride (EDC), 2-(N- Morpholino) ethanesulfonic acid (MES), and Cit were obtained from Aladdin Co. (Shanghai, China). DMEM, fetal bovine serum (FBS) and trypsin were acquired from Gibco. DAPI was purchased from Beyotime Institute of Biotechnology (Shanghai, China). Cell Counting Kit-8 (CCK-8) was purchased from Dojindo (Japan). The Annexin V-FITC & PI Apoptosis Kit was obtained from Ubio Biotechnology Pvt Ltd (Beijing, China). All other reagents were of analytical grade.

### Synthesis of carboxyl-functionalized MSN (MSN/COOH)

First, MSN were functionalized through the addition of carboxyl groups onto their surfaces by CPTS [44]. Briefly, 10 mg MSN was dissolved in 10 mL absolute ethyl alcohol, and 100 μL CPTS was added dropwise under stirring at 80°C in airtight conditions, followed by incubation for 24 h at room temperature. The samples were collected by centrifugation at 9000 rpm for 5 min, and then washed with ethyl alcohol to remove the excess CPTS. Finally, the precipitates were freeze-dried for further use.

### Combination of MSN/COOH with the TAT-FITC peptides (MSN/COOH/TAT- FITC)

Samples of MSN/COOH (10 mg) were dissolved in 10 mL isotonic MES saline buffer (0.1 M, pH 5.5), and 10 mg each of EDC and sulfo-NHS were added into the solution under stirring (300 rpm) at room temperature. After 2 h, the mixture was centrifuged at 9000 rpm for 5 min and washed with ultrapure water to remove the excess EDC, sulfo-NHS and other by-products. The precipitates were collected and resuspended in 10 mL isotonic MES saline buffer (0.1 M, pH 8), and 1 mg TAT-FITC peptide was added under stirring (300 rpm) at room temperature for 24 h in the dark. The samples were retrieved by centrifugation (9000 rpm, 5 min), washed with ultrapure water and freeze-dried for further use.

### Incorporation of MSN/COOH/TAT-FITC with the YSA-BHQ1 peptides (MSN/COOH/TAT-FITC/Cit/YSA-BHQ1)

Samples of MSN/COOH/TAT-FITC (10 mg) were dissolved in 10 mL sodium bicarbonate (0.5 M, pH 9.0). Then 200 μL Cit was slowly dropped into the solution under stirring (300 rpm) at room temperature overnight. During the reaction, aqueous NaOH was added to keep the pH of the reaction solution above 8 [45]. The samples were collected by centrifugation at 9000 rpm for 5 min, washed with ultrapure water and redispersed with 10 mL PBS buffer (pH 7.4). Subsequently, 1 mg YSA-BHQ1 peptide was added to the solution under stirring (300 rpm) at room temperature. After 8 h, the mixture was centrifuged at 9000 rpm for 5 min, washed with ultra-pure water to remove the redundant YSA-BHQ1 and freeze-dried for further use.

### Preparation of various DOX-loaded nano-particles

DOX molecules were encapsulated by the diffusion method [46]. Briefly, 10 mg samples of MSN/COOH, MSN/COOH/TAT-FITC, or MSN/COOH/TAT-FITC/Cit/YSA-BHQ1 was dissolved in 10 mL PBS buffer (pH = 7.4), then supplemented with 2 mg DOX, respectively. After being stirred (300 rpm) for 24 h, the mixture was centrifuged (9000 rpm) for 5 min, washed with PBS buffer to remove the excess DOX molecules, and freeze-dried for further use. The encapsulated DOX concentration was measured with a UV-Vis spectrophotometer (UV 722N, Shanghai Precision & Scientific Instrument Co. Ltd, China) at a wavelength of 485 nm. All processes were performed in the dark.

### Release behavior measurement of the DOX molecules

The *in vitro* release behavior of the DOX molecules from MSN/COOH/TAT- FITC/Cit/YSA-BHQ1 was investigated in different PBS buffers (pH = 7.4, 6.0, or 5.0) at 37°C. Free DOX was used as a control. Samples of MSN/COOH/TAT-FITC/Cit/YSA-BHQ1/DOX (5 mg) were dispersed in 5 mL PBS buffer, and each sample was transferred into a dialysis bag (molecular weight cut-off = 7,000 Da). The bag was immerged into 200 mL of the corresponding PBS buffer at 37°C and shaken at 100 rpm. At the desired time point, 3 mL of the PBS buffer outside the dialysis bag was removed for analysis. The concentration of DOX molecules in the PBS buffer was detected with a UV-Vis spectrophotometer at a wavelength of 485 nm. The same volume of fresh PBS buffer was then added for further experiments.

### Cell culture

MCF-7 and HEK293 cells were purchased from the Institute of Biochemistry and Cell Biology of the Chinese Academy of Sciences (IBCB, Shanghai, China). MCF-7 cells were chosen for the experiments because they overexpress the EphA2 receptor, which can specifically bind to the YSA peptide. Cells were cultured in DMEM/high-glucose medium containing 10% FBS, 100 U/mL penicillin G sodium and 100 μg/mL streptomycin sulfate at 37°C and 5% CO_2_ in a humidified incubator. The cell culture medium was renewed every 48 h. For all experiments, cells were routinely harvested for measurement. The number of cells was calculated with a hemocytometer (Perlong Medical Instruments Inc., Nanjing, China).

### *In vitro* cytotoxicity

The *in vitro* cytotoxicities of nano-particles or DOX-loaded nanoparticles were assessed by the standard CCK-8 test. MCF-7 and HEK293 cells were seeded in a 96-well plate (1 × 10^4^ cells/well) for 24 h and then incubated with the blank (culture medium without nano-particles), free DOX or DOX-loaded nano-particles at different concentrations. After incubation for 24 h, the culture medium was replaced with fresh medium containing 200 μL of 10% CCK-8 per well at 37°C for 2 h. The number of viable cells was measured at a wavelength of 450 nm with an Automated Microplate Reader Infinite F50 (Tecan Group Ltd, Switzerland). The cytotoxicity was assessed by the percentage of viable cells relative to the blank control.

### Cell apoptosis

The relative percentage of apoptotic cells was detected by flow cytometry. Briefly, MCF-7 and HEK293 cells were cultured in a six-well plate at an initial density of 2 × 10^4^ cells/cm^2^ and treated with free DOX, MSN/COOH/DOX, MSN/COOH/TAT- FITC/Cit/YSA-BHQ1/DOX, or MSN/COOH/TAT-FITC/Cit/YSA-BHQ1 nano-particles (equivalent DOX concentration of 5 μg/mL) for 24 h, separately. The cells were then harvested, washed twice with ice-cold PBS buffer, stained with Annexin V-FITC and PI for 10 min in the dark, and analyzed on a FACSCalibur Flow Cytometer (Becton Dickinson Company, USA).

### Intracellular distribution of nano-particles

The cellular uptake and intracellular distribution of nano-particles in the MCF-7 and HEK293 cells was observed with a Confocal Laser Scanning Microscope (CLSM) (Olympus, Japan). Briefly, the cells were seeded in a culture dish (1 × 10^4^ cells/dish) which was dedicated for the CLSM. After 24 h, the culture medium was replaced with fresh medium containing free DOX, MSN/COOH/DOX, MSN/COOH/TAT-FITC or MSN/COOH/TAT-FITC/Cit/YSA-BHQ1/DOX (equivalent DOX concentration of 5 μg/mL) followed by incubation for another 2 h. Then, the culture medium was discarded, and the cells were washed three times with PBS buffer to remove the extracellular free DOX molecules or nano-particles. DAPI was then used to stain the nuclei, according to the manufacturer's instructions. Next, the cells were washed three times with PBS buffer to remove the extracellular DAPI and were observed with the CLSM. As a further determination of the involvement of the EphA2 receptors in the cellular uptake of the nano-particles, the MCF-7 cells were preprocessed with excess YSA peptides for 1 h. The cells were then washed with PBS buffer to remove the extracellular free YSA peptides, treated with MSN/COOH/TAT-FITC/Cit/YSA- BHQ1/DOX for 2 h, washed with the PBS buffer, stained with DAPI, and observed with the CLSM.

### The fluorescent pH sensitivity of MSN/COOH/TAT-FITC/Cit/YSA-BHQ1

The fluorescence of MSN/COOH/TAT-FITC/Cit/YSA-BHQ1 under different pH conditions was observed with a fluorescence microscope (Shanghai Bimu Instrument Co. Ltd, China). Briefly, 1 mg MSN/COOH/TAT-FITC/Cit/YSA-BHQ1 was dissolved in 5 mL PBS buffer (pH 7.4). Then the solution was equally divided into four tubes and the pHs were adjusted to 5.5, 6.5, 7.4, and 8.0, separately. For comparison, 0.2 mg MSN/COOH/TAT-FITC or MSN/COOH/TAT-FITC/Cit was dissolved in 1 mL PBS buffer (pH 7.4). Then, the fluorescence of each solution was observed with the fluorescence microscope. The fluorescence intensities of the solutions were measured with a Fluostar Optima fluorescence microplate reader (BMG Labtech GmbH, Ortenberg, Germany) at excitation and emission wavelengths of 490 nm and 530 nm, respectively.

### Characterization of the nano-particles

The shapes and sizes of the nano-particles were observed with a transmission electron microscope (TEM) (JEOL JEM-1200EX microscope, Japan). The infrared spectra were inspected with a Fourier transform infrared (FT-IR) spectrometer (Nicolet Co., USA). The zeta potentials of the nano-particles were measured with a Malvern zetasizer 3000 (England). Nitrogen adsorption-desorption isotherms were acquired with a Quantachrome Nova 3000 Brunauer-Emmett-Teller specific surface area analyzer (Quantachrome Instruments, USA) at −196°C. Prior to the adsorption experiment, the samples were outgassed under a vacuum at 40°C for 6 h. The surface areas of the nano-particles were calculated by the Brunauer-Emmett-Teller means. The pore size distribution was determined from the nitrogen adsorption isotherms through the Barrett-Joynere-Halenda method. Thermogravimetric analysis was performed with a thermogravimetry analyzer (Pyris 1, PerkinElmer Co., USA) at a heating rate of 10°C min^−1^ in a N_2_ atmosphere from 30°C to 800°C.

### Statistical analysis

All experiments were carried out in triplicate and the obtained data were expressed as the mean ± standard deviation (SD). Student's *t-test* was used for statistical analysis. *P* < 0.05 was considered statistically significant.

## CONCLUSIONS

**Figure 11:**
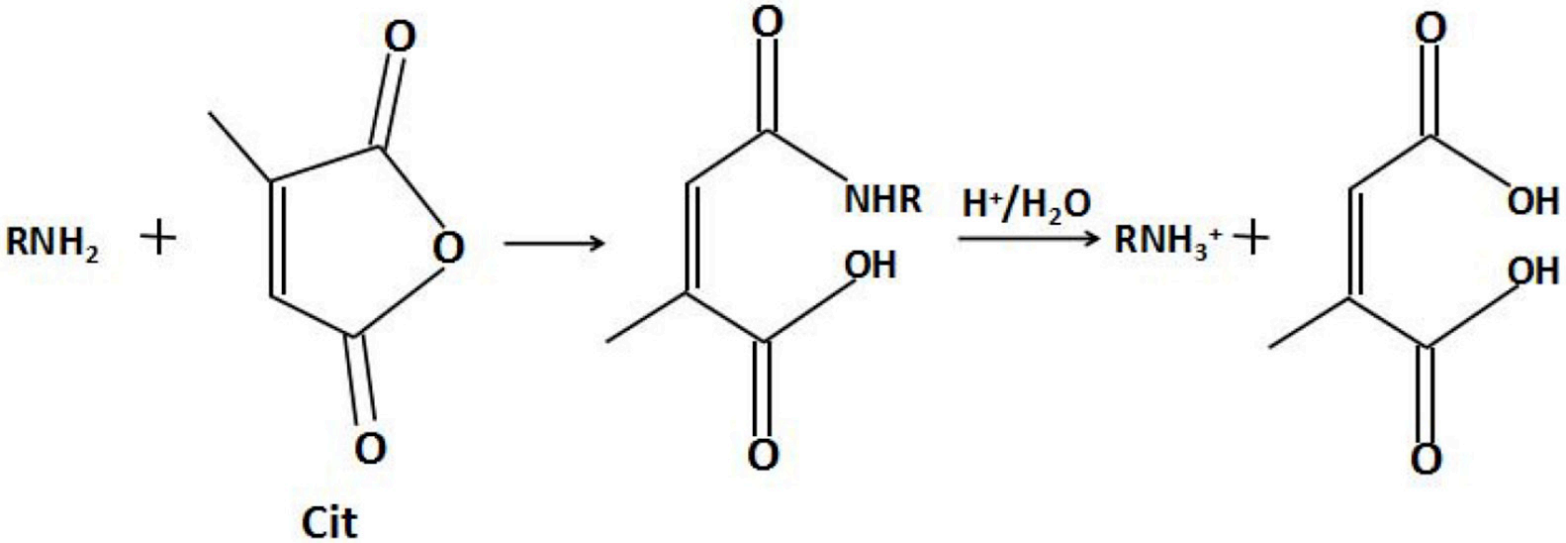
Schematic illustration of the charge inversion of a peptide amino

As illustrated in Scheme 1, the carboxyl groups were first modified on the surface of the MSN. Then, TAT peptides labeled with fluorescein isothiocyanate (FITC) were linked to the carboxyl groups. At this time, citraconic anhydride (Cit), an α-methyl derivative of maleic anhydride, was mixed with MSN/COOH/TAT-FITC in order to convert the primary amino groups of the TAT peptides into carboxyl groups by forming citraconic amides [47–49] (Figure 11). This was the first inversion of the TAT peptide charge (positive charge → negative charge). The resulting negatively charged TAT peptides could electrostatically bind with the positively charged YSA-BHQ1 peptides. The latter are short peptides that specifically bind to the EphA2 receptor, which is overexpressed in several cancer cell lines [33–35]. Finally, doxorubicin hydrochloride (DOX) molecules were trapped into the MSN. Due to the combination of the TAT and YSA peptides, the nuclear targeting activity of the TAT peptides was temporarily shielded. At the same time, the quenching group (BHQ1), which was used to tag the YSA peptides, closely approached FITC and quenched its fluorescence via fluorescence resonance energy transfer.

Citraconic amide is relatively stable in neutral and weak alkaline conditions, but is hydrolyzed readily in acidic media because the neighboring carboxylic acid group facilitates intramolecular catalysis, which regenerates its primary amine functionality [47, 48, 50–52] (Figure 11). Therefore, the positive charge of the TAT peptide was restored in this second charge reversal (negative charge → positive charge). Due to charge repulsion, the YSA peptides on the nano-particles separated from the TAT peptides. Thus, the TAT peptides (which were previously shielded by the YSA peptides) were exposed again, the nuclear targeting potential was restored, and FITC reemitted fluorescence. The TAT peptide then could combine with the nuclear pore complex on the karyotheca to form the transport complex, nano-particle/importin α/β. Subsequently, the drug carrier nano-particles were transferred into the nucleus. Lastly, the DOX molecules were released from the nano-particles, acted on the DNA double-strand, and caused the apoptosis of the tumor cells.

This nano-drug delivery vehicle had four notable features: 1. Active and passive targeting of tumor cells. The outermost YSA peptides could specifically bind to the EphA2 receptors, therefore facilitating the cellular uptake of the nano-particles. Meanwhile, due to the enhanced permeability and retention effect, the nano-drug-delivery vehicle could easily accumulate in the tumor cells. 2. pH-sensitive fluorescent probe. Because citraconic amide is stable in neutral and weak alkaline environments, the fluorescence of FITC was quenched by BHQ1. Thus, the fluorescence should be minimal in the blood circulation and normal tissues. On the other hand, in acidic conditions (like those of tumor tissues/cells), the citraconic amide was quickly hydrolyzed and the YSA peptides were repelled from the nano-particles, so FITC emitted fluorescence again; thus, the signal-to-noise ratio was maximized. 3. High-efficiency intranuclear drug delivery. The temporary masking of the positive charges of the TAT peptides should reduce their clearance from the circulation. Through this “Trojan Horse” method [9], the nano-particles should be safely escorted into tumor cells. 4. Simultaneous diagnostics and therapeutics. Fluorescence imaging can be used not only to assess tumor development, but also to determine the therapeutic performance of the drug-delivery system. Therefore, MSN/COOH/TAT-FITC/Cit/YSA-BHQ1/DOX could be a promising nano-drug delivery system for cancer imaging and therapy.
